# Coala: a standard-based framework for converting CWL-described command-line tools into agentic toolsets

**DOI:** 10.1093/bioinformatics/btag641

**Published:** 2026-08-26

**Authors:** Qiang Hu, Qianqian Zhu, Hong Zhang, Tao Liu, Song Liu

**Affiliations:** Department of Biostatistics and Bioinformatics, Roswell Park Comprehensive Cancer Center, Buffalo, NY 14203, United States; Department of Biostatistics and Bioinformatics, Roswell Park Comprehensive Cancer Center, Buffalo, NY 14203, United States; Department of Biostatistics and Bioinformatics, Roswell Park Comprehensive Cancer Center, Buffalo, NY 14203, United States; Department of Biostatistics and Bioinformatics, Roswell Park Comprehensive Cancer Center, Buffalo, NY 14203, United States; Department of Biostatistics and Bioinformatics, Roswell Park Comprehensive Cancer Center, Buffalo, NY 14203, United States

## Abstract

**Summary:**

Large Language Models (LLM) can orchestrate computational analyses through agentic systems, but scaling their toolsets remains a barrier because tool definitions are often hard coded into agent implementation. We developed Command-line LLM-agent Adapter (Coala), a standard-based framework that bridges the Model Context Protocol (MCP) and the Common Workflow Language (CWL). Coala turns CWL tool descriptions into MCP-compatible, LLM-accessible schemas, treating tool definitions as data rather than code. Tools are then executed in containerized environments through a generic MCP server, which separates the agent’s reasoning from tool execution. This framework improves reproducibility, reduces ongoing maintenance burden, and enables interactive access to local command-line tools through natural-language queries.

**Availability and implementation:**

Coala is available at https://coala.info and is openly developed on GitHub: https://github.com/coala-info/coala.

## 1 Introduction

Recent advances in Large Language Models (LLM) present an opportunity for computational biology, potentially enabling researchers to orchestrate computational analyses through natural language (Gao *et al.* 2024, Ren *et al.* 2025, Yang *et al.* 2025). However, moving from high-level reasoning to the actual execution of computational analysis remains challenging due to the operational reality of local computing environments, the scalability limits of hard-coded tool wrapping, and the critical need for scientific reproducibility.

First, the bioinformatics analysis often operates under constraints regarding both data and software deployment. Unlike general-purpose agents that rely on public web APIs, bioinformatics analysis is dominated by command-line utilities designed for local or high-performance computing (HPC) environments (da Veiga Leprevost *et al.* 2017, Gruning *et al.* 2018). This reliance on local execution is driven not only by the logistical challenges of transferring large datasets (e.g. BAM or VCF files) to external servers, but also by strict data governance requirements, licensing restrictions, and the convenience of leveraging local compute resources (Rahimzadeh *et al.* 2025). Consequently, an effective agentic framework must support local execution, interacting directly with deployed binaries rather than assuming a web service that is often unavailable in this domain.

Second, integrating these command-line tools into AI frameworks faces a maintenance bottleneck. While the Model Context Protocol (MCP) has emerged as a promising standard for connecting LLMs to external tools, it standardizes the transport layer rather than the content of tool definitions (Hou *et al.* 2025). Using MCP alone still requires developers to write custom code (e.g. Python wrappers) to define the inputs, outputs, and command structures for every individual tool. Given the vast and rapidly evolving bioinformatics utilities, this custom coding approach is not efficient for agent maintenance. Without a standardized, community-validated description language to accompany the connection protocol, integration remains ad hoc, requiring significant effort to expose even a small set of available tools.

Third, relying on LLM-generated scripts or non-standardized local environments compromises reproducibility. Agents that generate free-form scripts or run commands in an undefined local environment can introduce run-to-run variability. An LLM may hallucinate invalid command-line options or rely on machine-specific dependencies, leading to workflows that function on one machine but fail on another (Ji *et al.* 2023, Chen *et al.* 2025). For AI-driven analysis to be suitable for peer-reviewed research, it must yield auditable outputs and portable execution logs that adhere to the standards of reproducible science (Stodden *et al.* 2016, Trisovic *et al.* 2022).

To address these challenges, we introduce a standardized framework, Coala (Command-line LLM-agent Adapter), that bridges the MCP with the Common Workflow Language (CWL), a standard that specifies tools and workflows with validated schemas and containerized environments (Crusoe *et al.* 2022). By wrapping a CWL execution engine within a generic MCP server, we decouple the agent’s reasoning capabilities from the technical details of tool execution. This approach addresses the locality constraint by deploying a local MCP server to expose an interface for running local command-line tools on-site; it mitigates the maintenance bottleneck by leveraging existing community repositories of CWL tool descriptions to populate tool definitions (Hu *et al.* 2021, Yuen *et al.* 2021, Gustafsson *et al.* 2025), while simplifying the integration of new tools via declarative configuration rather than custom code; and it facilitates reproducibility by enforcing a declarative execution model where analyses are run in Docker containers.

## 2 Implementation

**Figure 1:**
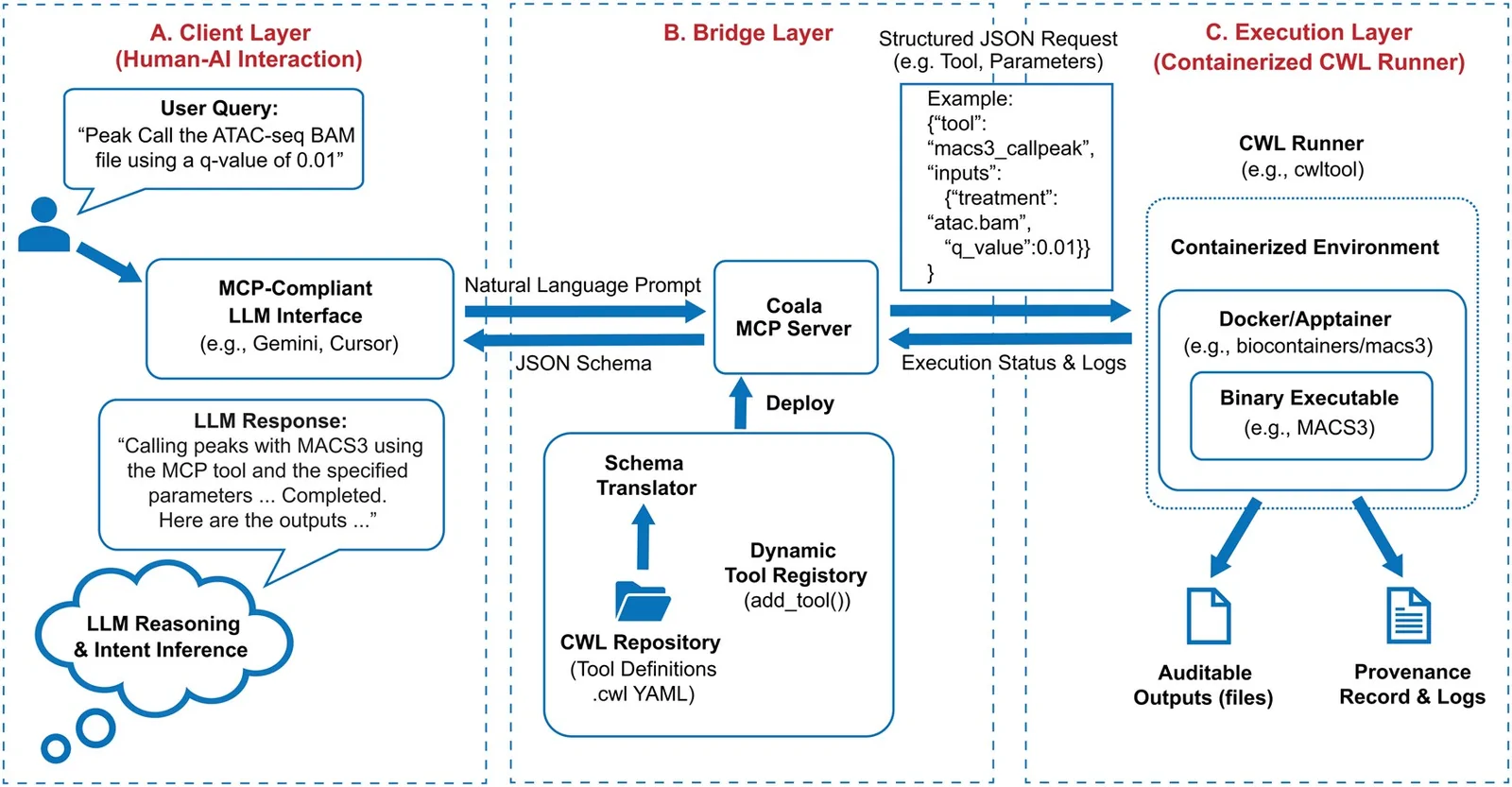
Overview of Coala architecture and data flow. Coala uses a three-tier design to translate natural-language queries into reproducible tool execution. (A) Client layer: An MCP-compliant client captures user queries. The underlying LLM infers intent and selects tools by matching requests to schemas exposed by the bridge layer. (B) Bridge layer: A local, generic MCP server parses CWL tool definitions (YAML) and generates MCP-compatible tool schemas (JSON) for discovery and invocation. A tool registry function lets users scope the active toolset to the task, improving selection accuracy and reducing token overhead. (C) Execution layer: After the LLM issues a tool call, a standard CWL runner (e.g. cwltool) executes the underlying binaries within containerized environments (e.g. Docker/Apptainer), producing standardized provenance (e.g. container hashes and exact command-line arguments) to support reproducibility across computing environments.

The proposed framework, Coala, operates on a three-tier architecture including a client layer, a bridge layer, and an execution layer. The client layer consists of an MCP-compliant client application (e.g. Claude Code, Codex, Gemini CLI, or Cursor) that utilizes LLMs (such as Gemini, GPT-5, or Claude) to enable natural language interaction. The bridge layer is a local generic MCP server that acts as a schema translator. Unlike standard MCP servers that require custom wrappers for each tool, the bridge layer automatically parses CWL definitions and exposes CWL-described command-line tools as executable MCP tools. It does not contain a hard-coded wrapper for each tool; rather, it performs generic schema mapping that reads the CWL definition (YAML) and translates it into the MCP-compatible JSON Schema format. The Execution Layer consists of a standard CWL runner (e.g. cwltool) to execute the underlying binaries [e.g. MACS3 (Wang *et al.* 2025)] within containerized environments. While Docker is commonly used for local workstations, standard CWL runners natively support Apptainer (formerly Singularity). This native support ensures that Coala can be deployed in HPC environments where Docker is typically prohibited due to security constraints. This ensures that analyses are reproducible and isolated from the host system’s dependencies. The interaction between these layers and the resulting data flow is illustrated in Fig. 1. A comparison of the Coala framework against the standalone MCP architecture and the baseline chatbot approach is summarized in Table 1.

**Table 1:** Comparison of interaction modes between LLM and local command-line tools.

	Baseline Chatbot model (No MCP, No CWL)	Custom-coded MCP agent (MCP only, No CWL)	Coala (MCP-CWL)	Other Pipeline Engines (e.g. Nextflow, Galaxy)
Architecture	Direct shell generation: The LLM generates raw commands to execute specific tools based on the user prompt	Custom wrapping: A local MCP server executes custom-coded functions that wrap specific tools	Schema translation: A generic local MCP server parses standard CWL definitions for tool execution, allowing conversational LLM orchestration.	Optimized for executing end-to-end pipelines; not natively designed for conversational LLM orchestration.
Tool definition	Risk of hallucination: LLM infers flags based on training data, which could be outdated or wrong (e.g. using MACS2 flags for MACS3).	Maintenance bottleneck: Users need to write and maintain a new wrapper to define inputs/outputs for every tool	Schema-driven: Tool interfaces are read directly from standard YAML files; New tools are added by swapping the .cwl file without code changes	Declarative tool abstraction layers exist (e.g. nf-core modules, Galaxy XML), but tightly coupled to their specific workflow engine.
Reproducibility	Uncontrolled: Results depend on the user’s local environment and installed versions.	Variable: Developers can manually hard-code container support (e.g. Docker dispatch) per tool, but it lacks a standardized, generalized declarative schema.	Enforced: Reproducible via Docker/Apptainer containers and explicit parameter bindings defined in the CWL	Reproducible via container integration (Docker/Apptainer) and parameter tracking.

Coala implements just-in-time tool retrieval to mitigate “context pollution” (Huang *et al.* 2026). This issue occurs when an LLM’s context window is overwhelmed by excessive or irrelevant information, degrading its reasoning and attention capabilities. Rather than scanning and loading an entire library of tool definitions upfront, the framework utilizes a tool registry function. This allows the user to selectively curate the agent’s capabilities for a specific task (e.g. loading only specified ATAC-seq analysis tools for a chromatin analysis session), minimizing token usage and reducing the probability of hallucinations by restricting the LLM’s action space to a relevant subset of tools.

## 3 Usage

The user first registers necessary tool definitions, which are either sourced from community CWL repositories or authored following the CWL Specification. The user sends natural language queries (e.g. “Peak call the ATAC-Seq BAM file using a q-value of 0.01”) to the MCP Client (e.g. Claude Desktop). The Client retrieves the tool list from the MCP server. The LLM selects the appropriate tool and sends a structured request for the analysis. Upon receiving the request, Coala translates this selection into a CWL job and executes it within a container (Docker/Apptainer). The execution logs and results are returned to the LLM, which interprets them and presents the final answer to the user. This ensures that while the LLM’s reasoning may be probabilistic, the resulting computational execution is reproducible. When an underlying tool fails (e.g. due to an incompatible parameter or wrong file format), the CWL runner produces a non-zero exit code. Coala captures the standard error log and passes it back to the LLM via the MCP interface, allowing the agent to troubleshoot the failure or notify the user. Four extended use cases are provided in the Coala website (https://coala.info/use-cases), where we demonstrate interactive, query-driven analysis for gene variants, RNA-Seq, ATAC-seq, and Hi-C data.

Worked Example: To demonstrate Coala’s query-driven analysis, we present two condensed interactions from the Hi-C use case.

- Interaction 1: Contact map. A user provides a multi-resolution .mcool file and asks to “show the contact map on chromosome 2 with 1Mb resolution.” The agent maps this to the “show” subcommand of the Cooler tool and translates the conversational request into the exact genomic coordinates required, structuring the payload as: {“uri”: “resolutions/1000000”, “range”: “chr2:0-242193529”}. Coala executes the CWL tool within Docker, returning a 242 × 242 contact matrix image.
- Interaction 2: TAD boundaries. The user subsequently requests to “Find the boundaries between TADs using 10kb resolution data… using window sizes of 100 kb and 200 kb, and apply the Li method.” The agent maps this to the “insulation” subcommand of the Cooltools tool, formatting the specific arrays and text flags into the JSON payload: {“uri”: “resolutions/10000”, “window”: [100000, 200000], “threshold”: “Li”}. Coala executes the CWL tool within Docker, returning a one row per 10kb dataframe file containing the resulting insulation scores and boundary flags.

We benchmarked Coala’s performance against an LLM chatbot across four use cases (Table S1). To ensure an objective comparison, both the Coala framework and the baseline chatbot were powered by the same underlying LLM (Gemini 3) and received identical natural language prompts. Coala achieved accurate task completion across all four use cases while requiring fewer tool call attempts and reducing token usage. The baseline chatbot failed in three of the four use cases, either due to unresolved package dependencies or generating custom fallback scripts that yielded incorrect results. A direct quantitative comparison with a custom-coded MCP agent is not applicable, as its execution performance varies depending on the quality of the developer’s specific implementation. The advantage of Coala over a custom MCP is not an increase in execution accuracy, but rather the mitigation of developer burden and the enforcement of a generic command-line tool standard.

## 4 Conclusion

Here, we introduce Coala, a standard-based framework for turning command-line tools into reproducible, agent-accessible utilities that support natural-language interaction. By shifting tool descriptions into the CWL and exposing them via the MCP, Coala enables LLM agents to discover and execute tools through structured interfaces while maintaining the containerized execution environments and traceable input/output provenance required for reproducible scientific research.

A barrier to scaling agentic toolsets has been the tight coupling of tool definitions with agent implementation. Agent frameworks relying on custom-coded wrappers create a maintenance bottleneck, where tool inclusion requires developers to write specific code to handle inputs, outputs, parameter mapping, and error states. Coala mitigates this bottleneck by treating tool definitions as data rather than code, as CWL describes command-line interfaces as structured data objects. Using CWL as an intermediate abstraction, the framework allows LLM agents to leverage the existing ecosystem of community-curated descriptors without modification to the agent’s core codebase.

While Coala effectively mitigates the need to write custom MCP wrappers, we acknowledge an inherent tradeoff: the framework relies on the availability of CWL tool descriptions. Community repositories such as bio-cwl-tools and Dockstore provide robust coverage for core bioinformatics domains, particularly in genomics (e.g. alignment, variant calling) and transcriptomics. However, newly published utilities or highly specialized scripts often lack immediate CWL definitions, requiring users to author the YAML descriptors themselves before agentic integration is possible. It is also necessary to distinguish Coala’s use of CWL from other workflow languages like Nextflow (nf-core) (Di Tommaso *et al.* 2017) or Galaxy (Galaxy Community 2022). While Nextflow (nf-core) and Galaxy are optimized for executing end-to-end pipelines, CWL excels at describing individual command-line tools, which allows the LLM to act as the workflow engine itself, chaining tools dynamically based on user prompts.

Future development will focus on expanding this framework as a step toward bridging the communities of CWL and MCP. We are committed to implementing more user-oriented utility functions and engaging with open-source initiatives such as BioContextAI (Kuehl *et al.* 2025) with the goal of enhancing the reusability of community-contributed command-line tools for agent-supported biomedical research.

## Supplementary Material

btag641_Supplementary_Data

## Data Availability

There are no new data associated with this article.
