# Supplementary material for: Coala: a standard-based framework for converting CWL-described command-line tools into agentic toolsets
Source: Bioinformatics. 2026 Aug 26;42(9):btag641. doi: 10.1093/bioinformatics/btag641 (PMC13549935; doi:10.1093/bioinformatics/btag641)
Supplement: Supplementary file 1 [file btag641_supplementary_data.pdf]

Supplemental Table S1. Comparison of Coala and Chatbot in the four use cases

	Coala		Chatbot	
	# of tool call attempts	analysis status	# of tool call attempts	analysis status
**Gene Variant Analysis**				
Retrieve and Extract Variants	3	accurate	9	accurate
Filter Pathogenic Variants	2	accurate	1	accurate
Detailed Variant Analysis	1	accurate	4	accurate
Total # of tool call attempts	6		14	
Token Usage	102,754		211,720	
**RNA-Seq Analysis**				
Download GEO Dataset	2	accurate	8	accurate
Differential Expression Analysis	1	accurate	36	accurate
Download MSigDB Gene Sets	1	accurate	3	accurate
Gene Set Enrichment Analysis	1	accurate	12	inaccurate[a]
Generate Enrichment Plot	1	accurate	2	inaccurate
Total # of tool call attempts	6		61	
Token Usage	80,030		1,448,328	
**ATAC-Seq Analysis**				
Call and Summarize Peaks	2	accurate	10	accurate
Refine Peak Cutoff	2	accurate	1	accurate
Annotate Called Peaks	1	accurate	5	inaccurate[b]
Visualize Peak Regions	1	accurate	2	inaccurate
Total # of tool call attempts	6		18	
Token Usage	89,164		318,267	
**Hi-C Analysis**				
Generate Contact Map	6	accurate	8	inaccurate[c]
Calculate Expected Cis-Contacts	3	accurate	3	inaccurate
Generate Compartment Profiles	3	accurate	9	inaccurate
Generate Saddle Plot	4	accurate	1	inaccurate
Call TAD Boundaries	3	accurate	3	inaccurate
Total # of tool call attempts	19		24	
Token Usage	151,734		251,781	

[a]: The chatbot failed to install the fgsea package, and instead wrote custom R scripts to perform over-representation analysis instead of gene set enrichment analysis.
[b]: The chatbot attempted to use bedtools to intersect with gene annotation directly, resulting in incorrect annotation.
[c]: The chatbot failed to execute Cooler/Cooltools, and instead wrote python scripts to perform analysis, resulting in incorrect outcomes.
